# ADAR RNA editing on antisense RNAs results in apparent U-to-C base changes on overlapping sense transcripts

**DOI:** 10.3389/fcell.2022.1080626

**Published:** 2023-01-06

**Authors:** Riccardo Pecori, Isabel Chillón, Claudio Lo Giudice, Annette Arnold, Sandra Wüst, Marco Binder, Marco Marcia, Ernesto Picardi, Fotini Nina Papavasiliou

**Affiliations:** ^1^ Division of Immune Diversity, German Cancer Research Centre (DKFZ), Research Program Immunology and Cancer, Heidelberg, Germany; ^2^ Helmholtz Institute for Translational Oncology (HI-TRON), Mainz, Germany; ^3^ European Molecular Biology Laboratory (EMBL) Grenoble, Grenoble, France; ^4^ Department of Biosciences, Biotechnologies and Biopharmaceutics, University of Bari “Aldo Moro”, Bari, Italy; ^5^ Research Group “Dynamics of Early Viral Infection and the Innate Antiviral Response,” German Cancer Research Centre (DKFZ), Research Program Infection, Inflammation and Cancer, Division Virus Associated Carcinogenesis (F170), Heidelberg, Germany

**Keywords:** ADAR, RNA editing, U-to-C, MultiEditR, DDX58/RIG-I, LINC-P21

## Abstract

Despite hundreds of RNA modifications described to date, only RNA editing results in a change in the nucleotide sequence of RNA molecules compared to the genome. In mammals, two kinds of RNA editing have been described so far, adenosine to inosine (A-to-I) and cytidine to uridine (C-to-U) editing. Recent improvements in RNA sequencing technologies have led to the discovery of a continuously growing number of editing sites. These methods are powerful but not error-free, making routine validation of newly-described editing sites necessary. During one of these validations on *DDX58* mRNA, along with A-to-I RNA editing sites, we encountered putative U-to-C editing. These U-to-C edits were present in several cell lines and appeared regulated in response to specific environmental stimuli. The same findings were also observed for the human long intergenic non-coding RNA p21 (hLincRNA-p21). A more in-depth analysis revealed that putative U-to-C edits result from A-to-I editing on overlapping antisense RNAs that are transcribed from the same loci. Such editing events, occurring on overlapping genes transcribed in opposite directions, have recently been demonstrated to be immunogenic and have been linked with autoimmune and immune-related diseases. Our findings, also confirmed by deep transcriptome data, demonstrate that such loci can be recognized simply through the presence of A-to-I and U-to-C mismatches within the same locus, reflective A-to-I editing both in the sense-oriented transcript and in the cis-natural antisense transcript (cis-NAT), implying that such clusters could be a mark of functionally relevant ADAR1 editing events.

## 1 Introduction

Recent years have seen an exponential increase in RNA sequencing (RNA-seq) technologies providing scientists with an incredible amount of transcriptomic data. Once compared to genomic data (DNA-seq), RNA-seq reveals information about several post-transcriptional processes that RNA molecules can undergo. For example, RNA editing is a mechanism that alters the RNA sequence itself. In mammals, two distinct kinds of RNA editing have been described so far, adenosine to inosine (A-to-I) and cytidine to uridine (C-to-U). These edits are the result of the deamination activity by proteins belonging to the adenosine deaminase acting on RNA (ADAR) (Bass, 2002; Nishikura, 2010; Savva et al., 2012) and the apolipoprotein B mRNA editing enzyme catalytic subunit (APOBEC) (Blanc and Davidson, 2010; Sharma et al., 2015, 2019; Lerner et al., 2018; Pecori et al., 2022) families, respectively. Reverse transcriptase incorporates guanosines (G) and thymidines (T) into cDNA at positions where inosines and uridines are present in the RNA, leading to base changes not present in the genomic DNA. For this reason, editing sites can be detected by directly comparing RNA-seq to DNA-seq data or a reference genome (Levanon et al., 2004; Picardi and Pesole, 2013; Wang et al., 2016; John et al., 2017; Piechotta et al., 2017).

Several bioinformatics pipelines have been developed for the analysis of next-generation sequencing (NGS) data to detect RNA editing sites (Ramaswami and Li, 2016; Eisenberg and Levanon, 2018; Diroma et al., 2019), leading to a constant increase of entries in their catalogs and the generation of new databases (Kiran and Baranov, 2010; Ramaswami and Li, 2014; Picardi et al., 2017; Mansi et al., 2021). Despite these improvements, RNA editing detection in NGS datasets remains challenging due to the many sources of DNA-RNA sequence mismatches, leading to the necessity of routine validation by reverse transcription-polymerase chain reaction (RT-PCR). RT-PCR is a two-step method in which the RNA is first retrotranscribed into cDNA, and then cDNA is amplified at specific locations *via* PCR. This method has some variations; for example, cDNA can be produced from oligo-dT, random hexamers, or specific primers for a particular transcript. In this latter case, and when a Hot Start DNA Polymerase is used, the reverse transcription and PCR amplification of a specific target take place one after the other in the same tube, in a so-called one-step RT-PCR reaction. This method allows a fast and easy RT-PCR setup, optimal for RNA editing detection validation. Additionally, one-step RT-PCRs exclusively generate cDNA from the transcript of interest leading to higher sensitivity in RNA editing detection when the transcript of interest is poorly expressed or edited (Wacker and Godard, 2005; Kluesner et al., 2021).

In this study, we report the observation of a putative U-to-C RNA editing while validating some A-to-I ADAR1 editing sites. U-to-C edits were observed on an mRNA (*DDX58*) and a long intergenic non-coding RNA (*hLincRNA-p21*) nearby A-to-I editing sites. In both cases, U-to-C editing appeared to be regulated upon specific stimulations a feature characteristic of RNA modifications. After looking for an RNA modification that could lead to this base change, we realized that U-to-C edits result from A-to-I editing on overlapping antisense RNAs that had not been previously described. We have also confirmed this finding by the analysis of known sense–antisense transcripts through deep transcriptome data from human tissues.

## 2 Materials and methods

### 2.1 Cell lines, treatments, and transfections

RCK8 cells (DSMZ, Cat# ACC-561, RRID: CVCL_1883) and U2932 (DSMZ, Cat# ACC-633, RRID: CVCL_1896) were cultured at 37°C, 5% CO_2_, in RPMI-1640 medium (Sigma-Aldrich, Cat# R8758), supplemented with 15% fetal bovine serum (PAN Biotech, Cat# P40-37100) and 1% of Penicillin/Streptomycin (Sigma-Aldrich, Cat# P4333). A549 cells (DSMZ, Cat# ACC-107, RRID: CVCL_0023) were cultured at 37°C, 5% CO_2_ in high-glucose DMEM (Sigma-Aldrich, Cat# D6429) supplemented with 10% fetal bovine serum (PAN Biotech, Cat# P40-37100) and 1% penicillin/streptomycin (Sigma-Aldrich, Cat# P4333). HEK293T cells (obtained from DKFZ, ATCC, Cat# CRL-3216, RRID: CVCL_0063) were cultured at 37°C, 5% CO_2_ in high-glucose DMEM (Sigma-Aldrich, Cat# D6429) supplemented with 5% FBS (PAN Biotech, Cat# P40-37100) and 1% penicillin/streptomycin (Sigma-Aldrich, Cat# P4333). Cell lines were authenticated using Multiplex Cell Authentication by Multiplexion (Heidelberg, Germany) as described recently (Castro et al., 2013). Additionally, the purity of cell lines was validated using the Multiplex cell Contamination Test by Multiplexion (Heidelberg, Germany) as described recently (Schmitt and Pawlita, 2009). No Mycoplasma, SMRV or interspecies contamination was detected.

For interferon-alpha (IFNα) stimulation, 2.5 × 10^5^ HEK293T cells were seeded in 12-well plates in a total volume of 1 ml media containing 200 U/ml of IFN-α (PBL Assay Science, Cat# 11100–1). After 16 h, cells were collected, and RNA was extracted using a Qiagen RNeasy Plus kit (Qiagen, Cat# 74134).

For doxorubicin treatment, 10^5^ HEK293T cells were seeded in 24-well plates to have around 30%–50% confluency the day after. The following day the cells were transfected with pcDNA3-hLincRNAp21-MS2 (Chillón and Pyle, 2016) using a mix of plasmid DNA and polyethyleneimine (PEI, Polysciences, Cat# 23966) in an approximately 1:1 ratio (2.5 µg DNA:2 µg of PEI). 6 h post-transfection, the media was replaced with new complete media and 2 µM doxorubicin hydrochloride (Sigma-Aldrich, Cat# D1515) or DMSO only as control (Sigma-Aldrich, Cat# D2650) were added 10–12 h post-transfection, for 12 h. RNA was then extracted using a Qiagen RNeasy Plus kit (Qiagen, Cat# 74134).

### 2.2 Plasmids

pcDNA3-hLincRNAp21-MS2 contains the 3898 nt-long LIsoE2 isoform of the human lincRNA-p21 (GenBank: KU881768.1) tagged with 24 copies of MS2 RNA hairpins, as previously described (Chillón and Pyle, 2016).

LentiCRISPRv2 was a gift from Feng Zhang (Addgene, plasmid #52961; https://addgene.org/52961; RRID: Addgene_52961) (Sanjana et al., 2014). DNA oligos #12–13 were cloned into this plasmid following the instructions of “lenti-CRISPRv2 and lentiGuide oligo cloning protocol” (Addgene plasmid #52961) to generate lenti-CRISPR-ADAR1 exon 4 [from Pestal et al. (2015); Supplementary Figure S7A]. As a control, lenti-CRISPR-NT (Lenti-NT) was cloned accordingly using oligos #14–15 based on control 800 from the GeCKO v2 library (Sanjana et al., 2014). pCMVDR8.91 (coding for HIV gag-pol) and pMD2.G (encoding the VSV-G glycoprotein) were a kind gift from Prof. Didier Trono (Lausanne, Switzerland).

pSpCas9(BB)-2A-GFP (PX458) was a gift from Feng Zhang (Addgene plasmid # 48138; https://n2t.net/addgene:48138; RRID:Addgene_48138) (Ran et al., 2013). DNA oligos #16–19 were cloned into this plasmid linearized by restriction digestion (BbsI) using NEBuilder® HiFi DNA Assembly Master Mix (NEB, Cat# E2621). We, therefore, obtained three plasmids for knocking out human DTWD1, DTWD2, or TSR3 as previously described (Takakura et al., 2019; Babaian et al., 2020) and an additional non-targeting control (NT-ctrl) based on control 800 from the GeCKO v2 library (Sanjana et al., 2014).

### 2.3 Genome-wide A-to-I sense-antisense RNA editing analysis

Ribo-depleted RNA-seq experiments from seven human tissues (Supplementary Material S1) were selected from the “RNA Atlas” project (Lorenzi et al., 2021) and downloaded from GEO under the accession GSE138734. Known annotations for antisense and protein-coding genes were obtained from Gencode (v38), downloaded in gtf format, and converted into bed format. Antisense and protein-coding annotations were intersected by means of the “intersect” function embedded in the Bedtools package (Quinlan, 2014), discarding overlapping intervals less than 300 bp. The resulting genomic coordinates of overlapping sense-antisense genes were used as input in a modified version of REDItools (Picardi and Pesole, 2013), able to split reads according to their orientation. Only editing candidates supported by more than five reads and organized in non-redundant clusters (represented by A-to-G or T-to-C mismatches according to gene strandness) were retained. All the editing sites considered in this analysis are described in Supplementary Material S1.

Circular heatmaps were generated using the R package circlize (Gu et al., 2014) and the cytoband representation of the human genome assembly hg38. Heatmaps color scale represents an RPKM-like normalization of editing events.

The entire pipeline and scripts are available at https://github.com/BioinfoUNIBA/antisenseEditing.

### 2.4 A-to-I and U-to-C editing sites validation and quantification

For editing site validation, PCRs were performed on genomic DNA (gDNA) and RNA. gDNA was extracted using the High Pure PCR Template Preparation kit (Roche, Cat# 11796828001) following manufacturer instructions. PCR amplification was performed using Q5^®^ High-Fidelity DNA Polymerase (NEB, Cat# M0491). RNA was extracted using the RNeasy Plus Mini kit (Qiagen, Cat# 74134) and treated with DNase (Invitrogen, Cat# AM 1907). Following RNA extraction, RT-PCRs were performed with gene-specific primers (Supplementary Table S1) and a One-step RT-PCR kit (Qiagen, Cat# 210212). All the PCR products were purified (Macherey-Nagel, Cat# 740609) and analyzed by Sanger sequencing. Quantification of editing was performed directly from the Sanger traces using MultiEditR (Kluesner et al., 2021). Alternatively, the PCR products were cloned using a CloneJET PCR cloning kit (Thermo Scientific, Cat# K1232) according to the manufacturer’s instructions and transformed into DH5a bacteria (NEB, Cat# C2987). Ten to twenty resultant bacteria colonies were sent for sequencing to determine edits and their frequency in the targeted region. All the primers used in this study were designed using Primer-BLAST (Ye et al., 2012), AmplifX 2.0.7 (by Nicolas Jullien; Aix-Marseille Univ, CNRS, INP, Inst Neurophysiopathol, Marseille, France—https://inp.univ-amu.fr/en/amplifx-manage-test-and-design-your-primers-for-pcr) or ApE (by M. Wayne Davis, https://jorgensen.biology.utah.edu/wayned/ape/). The chromosomal locations of all the editing sites analyzed in this study are listed in Supplementary Table S2.

### 2.5 RT-qPCR

RNA was extracted using the RNeasy Plus Mini kit (Qiagen, Cat# 74134). Before qPCR, RNA was additionally treated with DNase (Invitrogen, Cat# AM 1907). cDNA synthesis was then performed using ProtoScript M-MuLV First-Strand Synthesis Kit (NEB, Cat# E6300) using 1 µg of RNA DNAse digested. cDNA was synthesized using oligo-dT or random primers to detect *DDX58* or *hLincRNA-p21*, respectively. Two microliters of a 1:2 diluted cDNA were used to set up a 10 µl qPCR reaction using SsoAdvanced Universal SYBR Green Supermix (Bio-rad, Cat# 1725270). Finally, fold change expression was calculated using the comparative CT method (ΔΔCT) (Livak and Schmittgen, 2001). Supplementary Table S1 lists all the primers used in this study.

### 2.6 Generation of a HEK293T ADAR1 knockout cell line

Lenti-CRISPR-ADAR1 or lenti-CRISPR-NT, in combination with pCMV-DR8.91 and pMD2.G, were calcium-phosphate transfected into HEK293T cells for lentiviral particle production (ratio 3:1:3). After 48–72 h, cell-free supernatant was collected and used for transduction of HEK293T cells. The transduced cells were selected with puromycin (1 μg/ml). As soon as non-transduced cells died (∼2 days), ADAR1 knockout cells were seeded in 96-well plates in a limiting dilution (0.5 cells/well). Upon expansion of single clones, ADAR1 KO clones were validated by Western blot (Cell Signaling Technology, Cat# 14175, RRID: AB_2722520) following IFN-α stimulation using β-Actin as a loading control (Sigma-Aldrich, Cat# A5441, RRID: AB_476744). Lenti-NT control cells were kept polyclonal. After screening, clones three and four were shown to completely abolish ADAR1 (p110 and p150) expression (Supplementary Figure S7B). Therefore, clone three was used for the experiments conducted in this work.

### 2.7 Generation of HEK293T DTWD1, DTWD2 or TSR3 knockout cell lines

pSpCas9(BB)-2A-GFP carrying the sgRNAs for DTWD1, DTWD2 or TSR3 were transfected into HEK293T cells using Lipofectamine 2000 (ThermoFisher, Cat# 11668019) following manufacturer instructions. 48 h after transfection, GFP-positive cells were sorted and plated (one cell per well) in 96-well plates. The clonality was validated by visual inspection with a microscope, and positive clones were screened by Sanger sequencing.

### 2.8 Statistical analysis and data visualization

Data were analyzed and plotted using GraphPad Prism (version 9.3.1). Specific information about data presentation is provided in each figure caption throughout the manuscript. Statistical significance was calculated by unpaired, two-tailed Student’s t-test: *p < 0.05, **p < 0.01, ***p < 0.001, ****p < 0.0001, ns: not significant.

## 3 Results

### 3.1 Observation of a persistent U-to-C base change in *DDX58*

**FIGURE 1:**
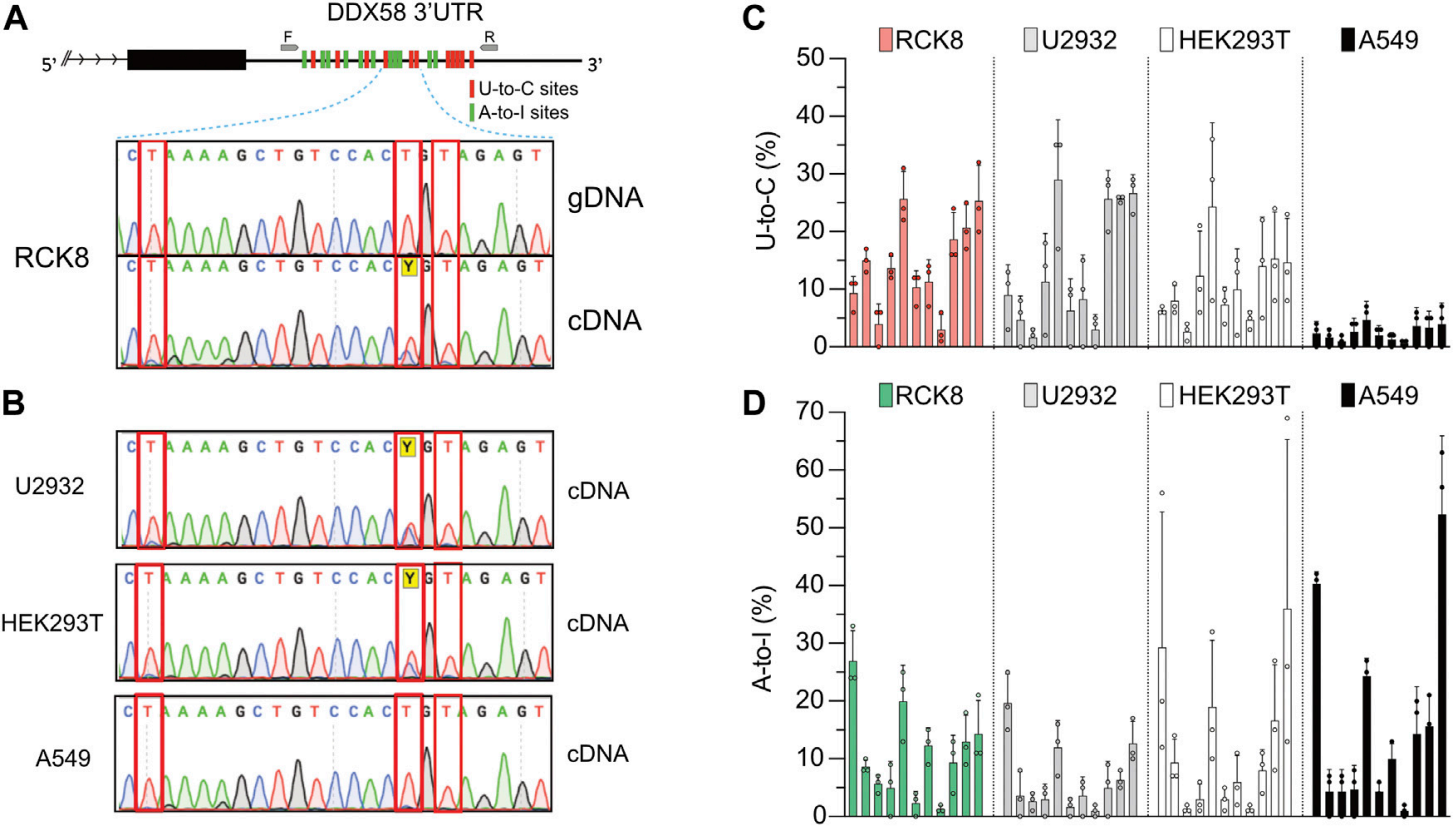
A persistent U-to-C base change in *DDX58* cDNA. **(A)** Upper: schematic representing the identification of 11 U-to-C (red bars) and 11 A-to-I (green bars) base changes within the 3′UTR of *DDX58* in the B cell line RCK8. Primers used for PCR amplification are indicated as small grey arrows. Lower: representative image of Sanger traces showing that U-to-C base changes (inside the red rectangles) are only present in cDNA and not in genomic DNA (gDNA). **(B)** These RNA base changes are present at the same in other cell lines. **(C,D)** Quantification of the 11 U-to-C **(C)** and 11 A-to-I **(D)** sites within different cell lines. Quantification was performed directly from Sanger traces using MultiEditR (Kluesner et al., 2021). Center = mean and error bars = standard deviation, N = 3.

RNA-seq data analysis represents a powerful method to detect new RNA editing sites. Unfortunately, these technologies are not error-free; thus, validation of these newly discovered RNA editing sites is still necessary. This validation is performed *via* PCR amplification of a specific region containing the editing sites to be validated from either DNA or cDNA (the latter represents the RNA). In a recent work from our lab, we identified RNA editing sites comparing RNA- and DNA-seq data in a cohort of Diffuse large B cell lymphoma (DLBCL) patients (Pecori et al., 2021). We used a one-step RT-PCR reaction to validate some of those sites due to its higher sensitivity in RNA editing detection for low edited or expressed transcripts (Wacker and Godard, 2005; Kluesner et al., 2021). While validating some A-to-I editing sites within the transcript *DDX58* in RCK8, a B cell lymphoma-derived cell line, we also observed the presence of numerous putative U-to-C edits. In a short region (∼600 nucleotides) of the 3′ untranslated region (3′UTR) of *DDX58* we could detect 11 A-to-I sites and 11 U-to-C sites (Figure 1A, upper). A-to-I and U-to-C RNA editing events are observed as A-to-G and T-to-C in cDNA. Despite all the detections and quantifications being done on cDNA, throughout this manuscript, we refer to them as A-to-I and U-to-C base changes.

All those edits are visible in Sanger sequencing following amplification of cDNA but not genomic DNA (gDNA), validating them as real RNA editing sites (Figure 1A, lower). While U-to-C editing is well described in plants (Yoshinaga et al., 1996; Knie et al., 2016; Ruchika et al., 2021), it has been rarely described in Metazoans (Villegas et al., 2002; Liu et al., 2004); thus, we decided to investigate further this preliminary observation. We then performed the same validation on another three cell lines, namely U2932, HEK293T, and A549. Except for A549, we confirmed the observation of putative U-to-C base changes at the same precise sites identified in RCK8 (Figure 1B). To check a possible functional connection between the A-to-I and U-to-C editing, we quantified the frequency of U-to-C and A-to-I at all sites for all the cell lines (Figures 1C, D). No specific trend was observed, with different cell lines showing variations in the level of both editing types. Altogether these findings demonstrate the presence of an apparent and persistent U-to-C RNA editing in *DDX58* mRNA. This editing can be found at the exact locations in different cell lines, and it seems independent of A-to-I editing. Indeed, the A549 cell line shows high A-to-I editing within *DDX58* but no U-to-C editing.

### 3.2 U-to-C editing in *DDX58* mRNA is dynamic

**FIGURE 2:**
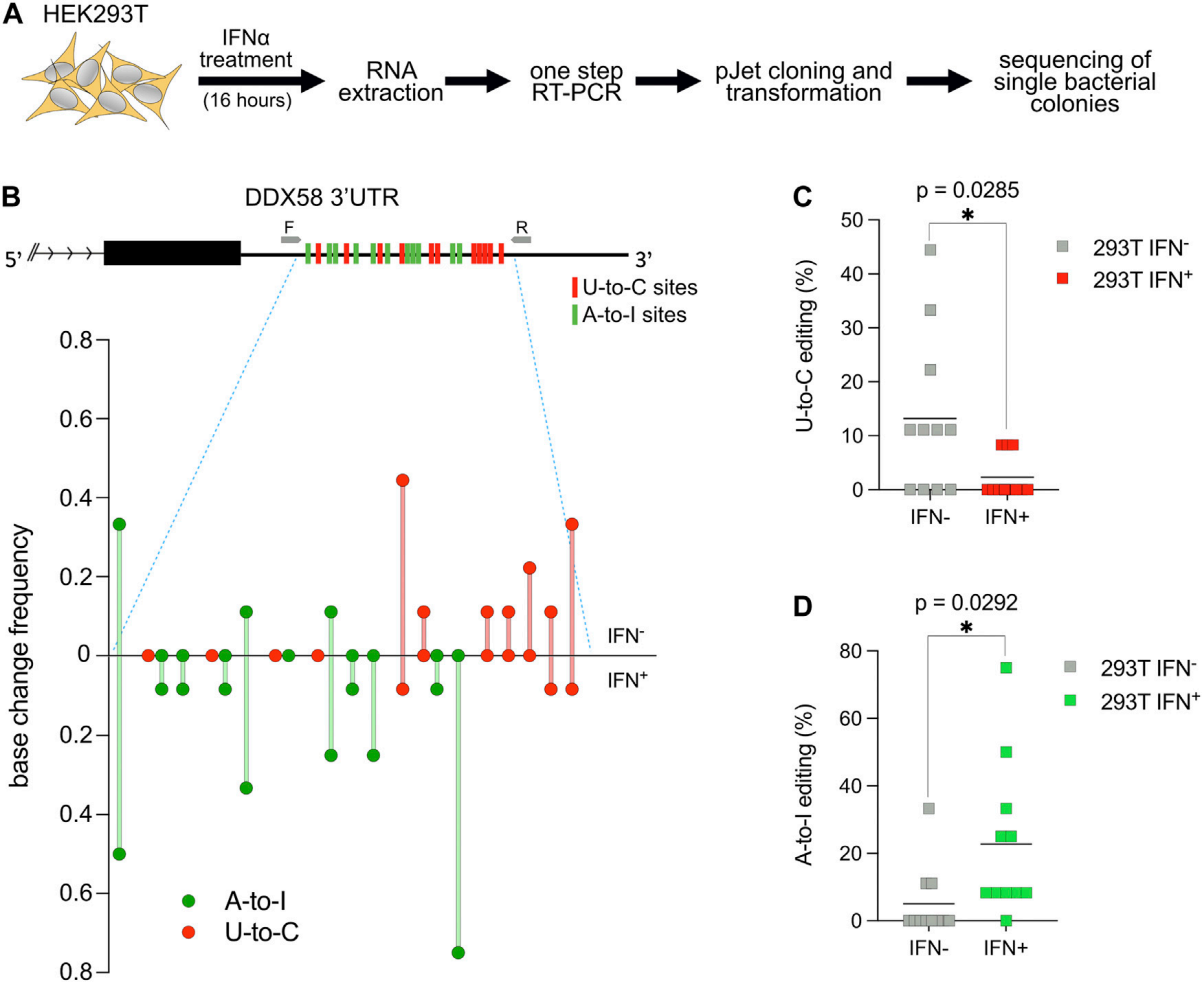
U-to-C base changes within DDX58 are dynamic. **(A)** Flowchart of the experiment. **(B)** Upper: schematic representing the 11 U-to-C (red bars) and 11 A-to-I (green bars) base changes within the 3′UTR of *DDX58*. Primers used for cDNA synthesis and PCR amplification are indicated as small grey arrows. Lower: Quantification of base changes within *DDX58* 3′UTR with and without interferon (IFN) treatment based on sequences from bacterial colonies (Supplementary Figure S2). U-to-Cs and A-to-Is are shown in red and green, respectively. **(C,D)** Dot plots showing the decrease in U-to-Cs and the increase A-to-Is upon IFN treatment. Each dot = one single site; line = mean. A two-tailed unpaired *t*-test was used to compare the differences (*p < .05).

It is known that RNA modification in general and RNA editing specifically are crucial during the cellular response to environmental stimuli or stress (Roundtree et al., 2017; Tan et al., 2017). To test if the U-to-C editing observed in *DDX58* would change after specific stimulation, we decided to treat HEK293T cells with interferon-alpha (IFN⍺). IFN⍺ treatment has two relevant consequences for this experiment: first, it induces ADAR1 p150 expression (Patterson et al., 1995), which leads to an increase in A-to-I RNA editing (Hartwig et al., 2004, 2006); second, it leads to the overexpression of *DDX58*, which is an interferon-stimulated gene (ISG) itself (Matsumiya and Stafforini, 2010). HEK293T cells were chosen for this experiment because of the high level of U-to-C editing observed within *DDX58* and because they are responsive to IFN⍺ stimulation (Figures 1B, C and Supplementary Figure S1). Following stimulation, RNA extraction and one-step RT-PCR were performed. PCR products were introduced into bacteria, and single bacterial colonies were sequenced using Sanger sequencing. Alignment to the unedited reference genome allowed us to easily count the editing sites in the presence or absence of stimulation to assess the frequency of A-to-I and U-to-C editing for each site in the two conditions (Figure 2A and Supplementary Figure S2). Not surprisingly, IFN⍺ stimulation leads to a significant increase in A-to-I editing (∼4-fold increase of the mean, Figures 2B, D) which is expected due to the induction of ADAR1 p150 expression (Patterson et al., 1995; Hartwig et al., 2004, 2006). However, the opposite effect was observed for U-to-C editing, for which the treatment led to a significant decrease (∼5-fold decrease of the mean, Figures 2B, C). These data suggest that putative U-to-C base changes are differently regulated compared to ADAR-induced A-to-I editing.

### 3.3 U-to-C editing within the long intergenic non-coding RNA *hLincRNA-p21*

After characterizing the U-to-C editing within *DDX58* mRNA, we asked if this editing was also present in other RNA species, such as long non-coding RNAs (lncRNAs). *LincRNA-p21* is a crucial molecule during the response to cellular stress driven by p53 (Huarte et al., 2010). While initially discovered in mice, *LincRNA-p21* is also present in humans (*hLincRNA-p21*, formally known as *TP53COR1*). Recent work has shown that hLincRNA-p21 contains inverted-repeat *Alu* elements, which can fold as independent domains (Chillón and Pyle, 2016). Interestingly, putative U-to-C editing events were identified in both sense and antisense *Alu* elements (Chillón and Pyle, 2016).

**FIGURE 3:**
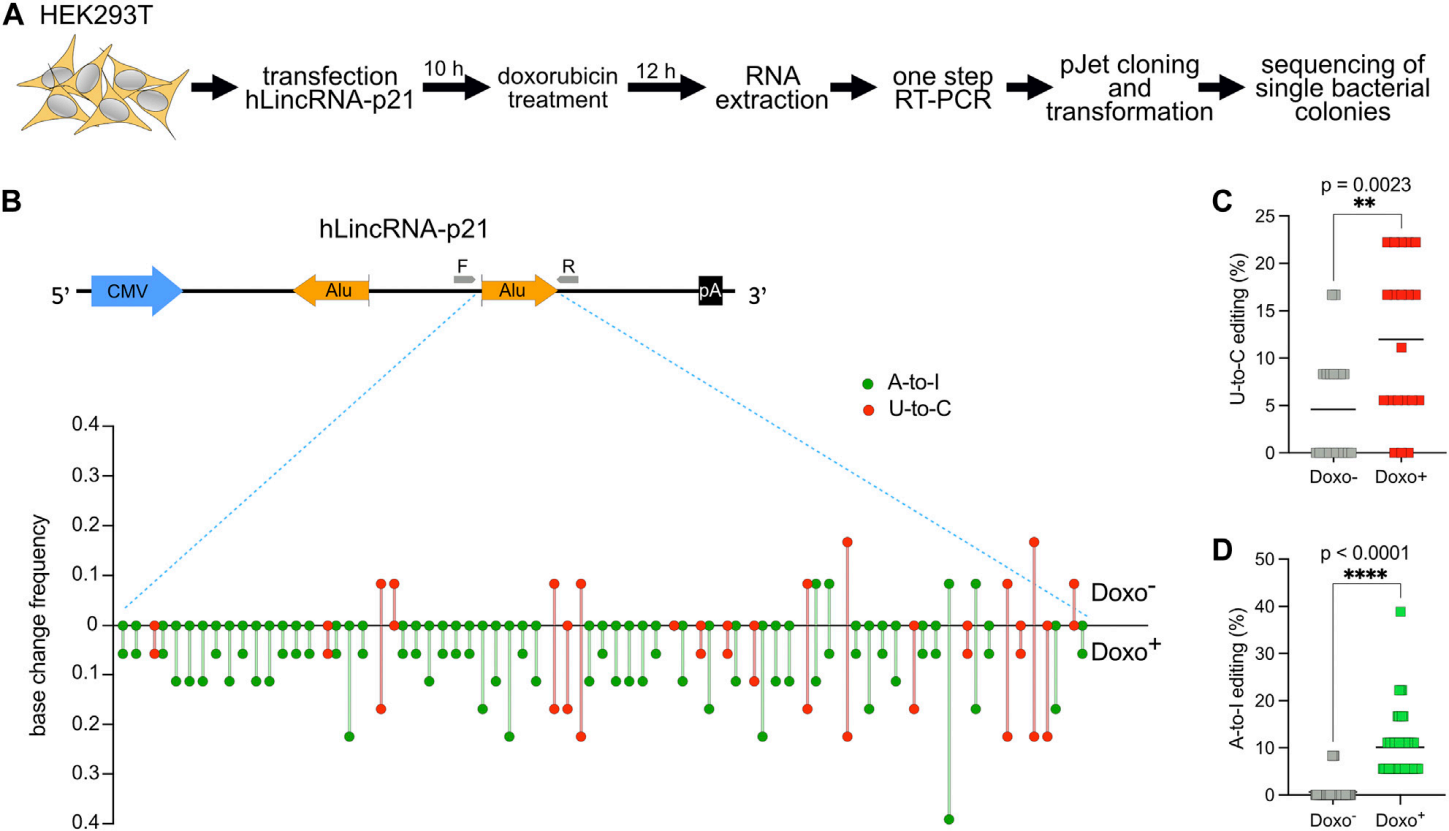
U-to-C base changes are present and dynamic also in the long intergenic non-coding RNA *hLincRNA-p21*. **(A)** Flowchart of the experiment. **(B)** Upper: schematic representing the plasmid used for overexpression of *hLincRNA-p21*. This long non-coding RNA contains two inverted Alu elements (big orange arrows). Primers used for cDNA synthesis and PCR amplification are represented as small grey arrows. Lower: Quantification of base changes within hLincRNA-p21 sense Alu element with and without doxorubicin (doxo) treatment based on sequences from bacterial colonies (Supplementary Figure S3). U-to-Cs and A-to-Is are shown in red and green, respectively. **(C,D)** Dot plots showing the increase of both U-to-Cs and A-to-Is upon doxorubicin treatment. Each dot = one single site; line = mean. A two-tailed unpaired *t*-test was used to compare the differences (**p < 0.01; ****p < 0.0001).

For this reason, we decided to first transfect in HEK293T a plasmid encoding *hLincRNA-p21* and then treat the transfected cells with doxorubicin, a chemotherapeutic drug that induces DNA damage. We then performed RNA extraction and one-step RT-PCR to amplify the sense *Alu*. Detection and quantification of editing were performed as described above for *DDX58* mRNA (Figure 3A). While doxorubicin treatment was shown to upregulate *hLincRNA-p21* in some cell lines (Chillón and Pyle, 2016), we did not observe any significant changes in the expression of the endogenous, or in the stability of the exogenous, *hLincRNA-p21* in HEK293T upon treatment (Supplementary Figure S3). In the absence of treatment, we observed only nine U-to-C and four A-to-I edits with editing frequency lower than 0.2 within the sense *Alu* (Figures 3B–D and Supplementary Figure S4). However, induction of DNA damage by doxorubicin leads to a significant increase in both editing types (∼2.5- and ∼16-fold increase of the mean for U-to-C and A-to-I, respectively; Figures 3B–D). Notably, we observed a substantial increase in the number of low-frequency (<0.1) edits that were not visible in the absence of stimulation (Figure 3B and Supplementary Figure S4). The increase of A-to-I editing upon DNA damage may be explained by recent findings showing an overall change in ADAR editing in response to DNA breaks (Jimeno et al., 2021). These data confirm the previous observation that putative U-to-C editing can also be identified in lncRNAs.

### 3.4 Apparent U-to-C base changes result from A-to-I antisense RNA editing

We then decided to look for the enzyme responsible for generating this U-to-C RNA editing. Few RNA modifications of uridines have been described to lead to a U-to-C base change. 4-thiouridine (s4U) itself leads to low levels of U-to-C transitions after reverse transcription (Hafner et al., 2010), and this level can be increased by chemical treatments of the RNA [reviewed in (Duffy et al., 2019)]. Indeed, s4U is often used in methods to study RNA metabolism because its presence can be easily detected *via* sequencing (Herzog et al., 2017; Schofield et al., 2018). Unfortunately, while s4U is present in bacterial and archaeal tRNAs, it has not been described in human tRNA (Boccaletto et al., 2018). It thus is very unlikely to be related to the U-to-C editing described here. In contrast, the 3-amino-3-carboxypropylation of uridine has been recently described in humans (Takakura et al., 2019). This modification leads to the formation of a 3-(3-amino-3-carboxypropyl) uridine (acp3U), which can be observed as an apparent U-to-C conversion caused by misincorporation during cDNA synthesis (Takakura et al., 2019; Kimura et al., 2020). Additionally, amino-carboxypropylation of methylated pseudouridine (ψ) has been described in rRNA in humans (Meyer et al., 2016). This m1acp3ψ modification perturbs standard base pairing during cDNA synthesis leading to U-to-C conversion (Babaian et al., 2020). Therefore, we decided to knock out the writers of these modifications, namely DTWD1, DTWD2, and TSR3, in HEK293T cells, as previously described (Takakura et al., 2019; Babaian et al., 2020). We successfully obtained knockout cell lines for those proteins. However, we did not observe any changes in U-to-C editing within *DDX58* (Supplementary Figure S5).

**FIGURE 4:**
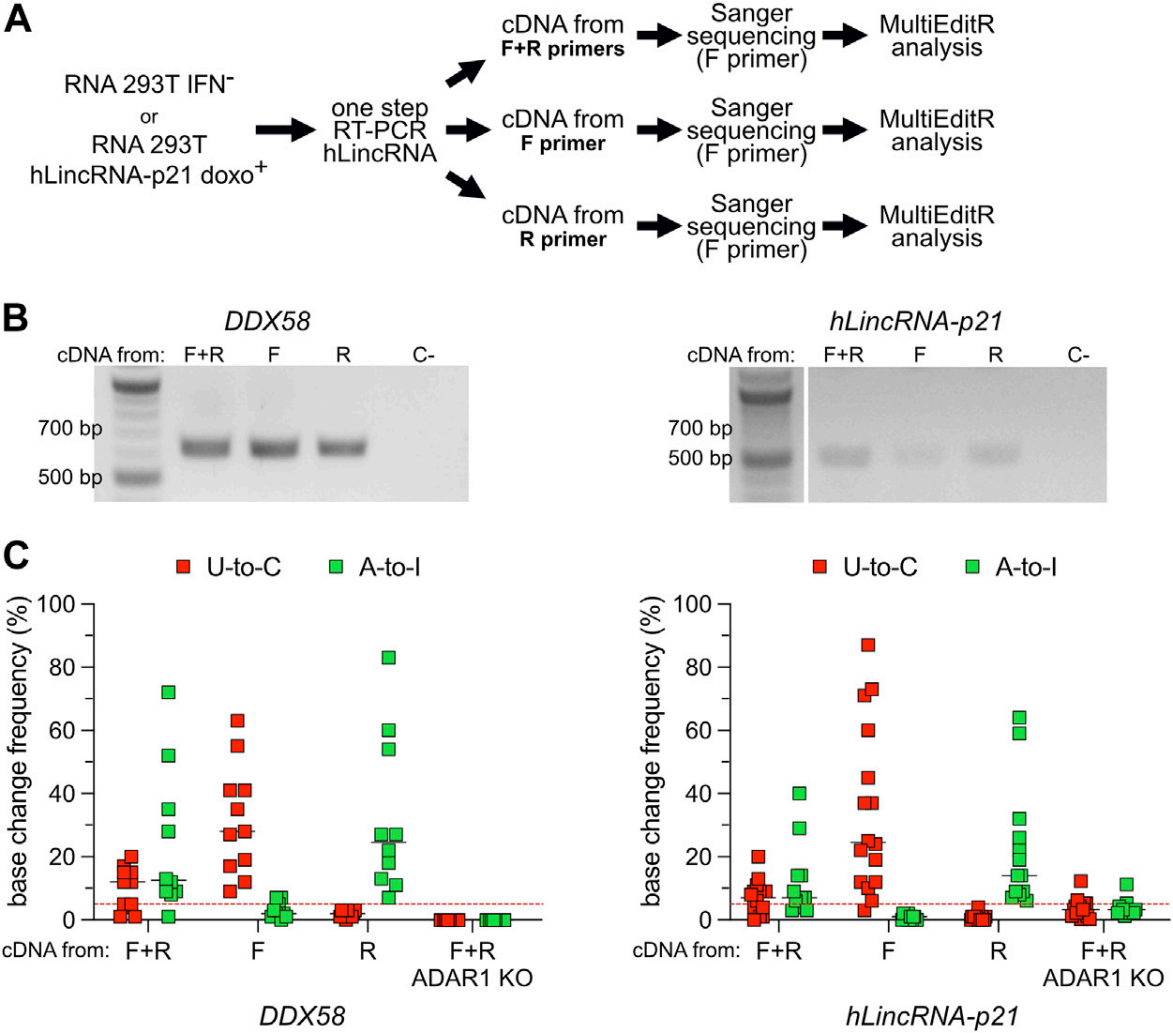
U-to-C base changes originate from A-to-I RNA editing on antisense RNA. **(A)** Flowchart of the experiment. **(B)** Representative agarose gel of the amplification products of *DDX58* and *hLincRNA-p21* upon one-step PCR using different primers for cDNA synthesis. C- = negative control. **(C)** Dot plots showing the editing frequency in U-to-Cs and A-to-Is measured from Sanger sequencing dependent on the primer used for cDNA synthesis. Only sites with editing higher than 5% in at least one condition are plotted. Each dot = one single site; line = median; red dashed line represents the limit of detection of MultiEditR (Kluesner et al., 2021). U-to-Cs and A-to-Is are shown in red and green, respectively.

A-to-I RNA editing has also been reported on antisense RNA, with some studies proposing that 15% of editing originated from transcripts expressed from the antisense strand (Porath et al., 2014). Widespread antisense transcription has been reported in humans, with 5%–10% of all genomic loci expressing overlapping sense and antisense RNAs (Lehner et al., 2002; Shendure and Church, 2002; Yelin et al., 2003). Overlapping sense and antisense RNAs often form structured motifs characterized by the presence of double-stranded hairpins that can act as substrates for ADAR (Supplementary Figure S6A). Using one-step RT-PCR methods with target-specific primers, the cDNA will be synthesized from both the sense and the antisense RNA. Following Sanger sequencing, A-to-I antisense RNA editing may result in an apparent U-to-C base (Supplementary Figure S6B). Therefore, we explored the possibility that previously-uncharacterized transcripts are expressed in antisense orientation to *DDX58* and *hLincRNA-p21* and modified by ADAR through A-to-I editing. To answer this question, we selected RNA samples from the two experimental conditions, which showed the majority of putative U-to-C editing in *DDX58* and *hLincRNA-p21*, namely IFN^−^ and doxorubicin^+^, respectively (Figures 2, 3). On these samples, we performed in parallel three different one-step RT-PCR, providing both forward (F) and reverse (R) primers, or only the F or only the R primer, during the cDNA synthesis step (Figure 4). In this way, we obtained strand-specific amplification, with F and R primers generating cDNA specifically from the antisense and sense RNA, respectively (Supplementary Figure S6B). Both, *DDX58* and *hLincRNA-p21* showed abundant amplification from the antisense RNA on an agarose gel (Figure 4B). Strikingly, antisense-specific amplification resulted in high detection of putative U-to-C and no detection of A-to-I. The opposite was observed following amplification of the sense RNA for both *DDX58* and *hLincRNA-p21* (Figure 4C). Additionally, standard one-step RT-PCR from a HEK293T ADAR1 KO cell line (Supplementary Figure S7) resulted in no U-to-C or A-to-I editing detected in *DDX58* and only a very low residual editing in *hLincRNA-p21* (Figure 4C). Our data demonstrate that the putative U-to-C editing results from A-to-I editing on the antisense RNA indicating high editing activity by ADAR1 on both sense and antisense *DDX58* and *hLincRNA-21.*

### 3.5 A-to-I antisense RNA editing in NGS

After observing antisense A-to-I RNA editing in both an mRNA and a lincRNA, we asked what the impact of this process at the transcriptome level in different tissues is. To elucidate this point, we investigated antisense A-to-I RNA editing genome-wide by using seven ribo-depleted strand-oriented RNA-seq experiments from various human tissues of the “RNA Atlas” project (Lorenzi et al., 2021). We created a catalog of sense-antisense gene overlaps based on Gencode annotations to provide an unbiased overview of antisense editing. Known antisense transcripts were initially selected from Gencode, then overlapping regions of at least 300 bp with sense transcripts were collected to a total number of 1677 suitable overlaps. For each one of these, corresponding to a well-defined genomic interval, we called RNA editing using pre-aligned RNA-seq reads and a modified version of the REDItools software (Picardi and Pesole, 2013) able to split reads according to their orientation. A-to-I RNA editing events supported by at least five reads and organized in clusters of A-to-G or T-to-C mismatches were selected for downstream analyses.

**FIGURE 5:**
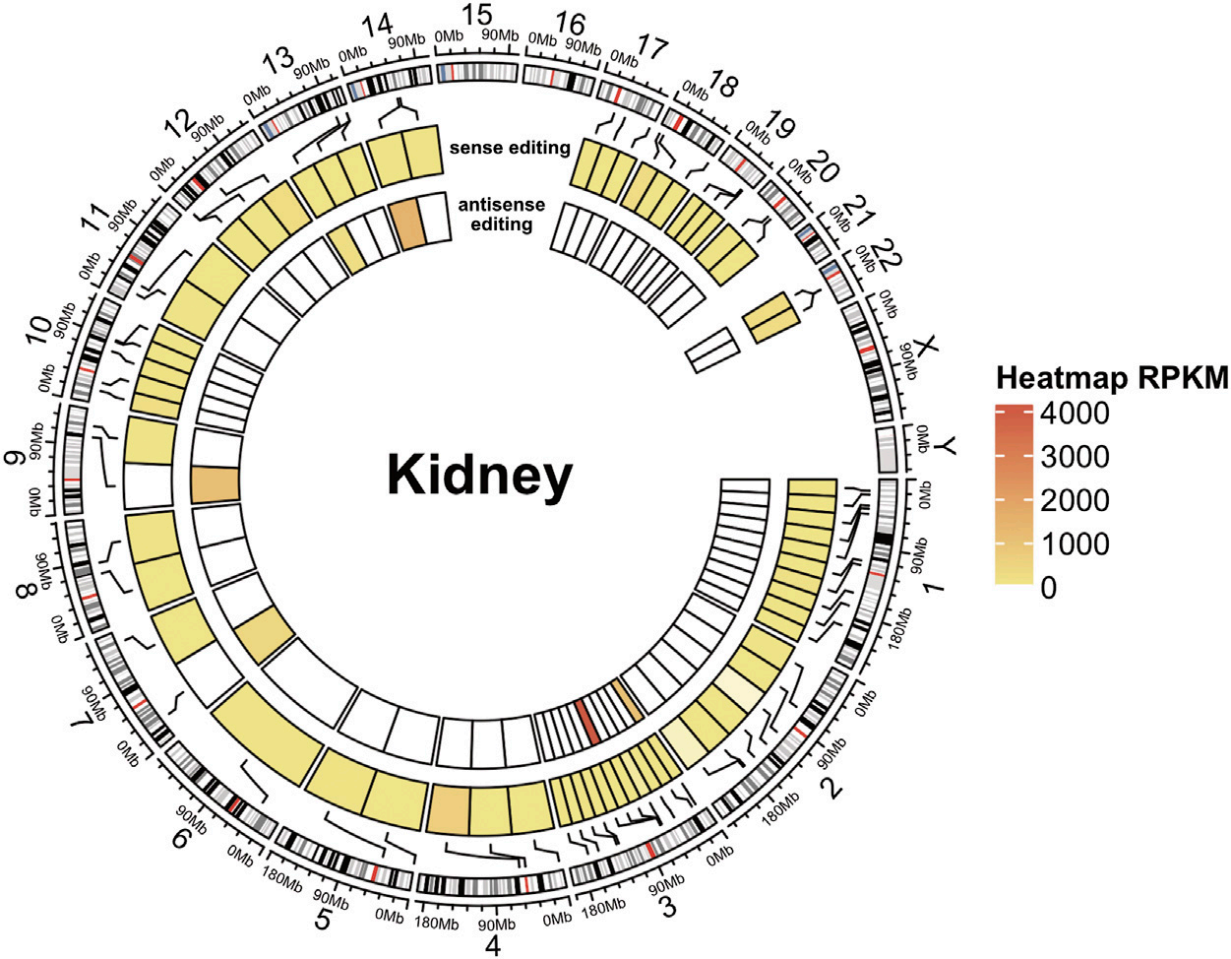
A-to-I antisense RNA editing in NGS data. Circular heatmap for a kidney RNA-seq sample. The external circle represents the chromosomes, and the two inner circles represent sense (intermediate circle) and antisense (internal circle) editing. A-to-I RNA editing locations are indicated with black lines connecting the heatmap to the cytoband context of the chromosomes (human genome assembly hg38). Editing levels are depicted in circular heatmaps using a color scale based on RPKM-like values.

On the whole, we observed that the number of A-to-I editing changes, normalized by the overlap length, was higher in the sense strands of overlaps than in antisense strands, and this trend was common to all analyzed samples and tissues, supporting the previous notion that antisense editing is low Figure 5 and Supplementary Figure S8; and (Neeman et al., 2005). On average, only 199 out of 1677 potential overlaps showed evidence of A-to-I RNA editing. Of these, 21 displayed obvious sense and antisense editing, 164 sense editing, and 35 antisense editing only.

However, DDX58 was not among the transcripts identified by our approach, suggesting its limitations. Namely, antisense transcripts might be less abundant, leading to lower read depth (and problems in detecting editing); alternatively, naturally poor editing on some transcripts might be reported as “no editing” (depending on cut-offs). Both of these are limitations to our approach. These limitations could be cell type-specific or disease-specific. Overall though, our work suggests that clusters of A-to-I (and U-to-C) editing might specify dually edited, convergently transcribed regions, offering a potentially simple way to identify loci that may be of disease relevance (Li et al., 2022).

## 4 Discussion

Recent improvements in RNA-seq and DNA-seq data have provided scientists with a considerable amount of data from which several new RNA editing sites were discovered. However, these technologies are also affected by other sources of DNA-RNA sequence mismatches. Thus, RNA editing detection from NGS data remains a challenging task (Ramaswami and Li, 2016; Eisenberg and Levanon, 2018; Diroma et al., 2019), and validation of newly discovered editing sites is necessary. Here, we report the observation of U-to-C base changes and A-to-I editing within *DDX58* mRNA and the lncRNA *hLincRNA-p21*. U-to-C edits show typical features of a *bona fide* RNA modification. Indeed, they can be identified in multiple cell systems and respond to environmental stimulation differently from other co-existing modifications. However, careful evaluation demonstrated that putative U-to-C corresponds to A-to-I editing introduced by ADAR on overlapping antisense transcripts (Figure 4). Antisense transcription is a frequent process within the human transcriptome (Lehner et al., 2002; Shendure and Church, 2002; Yelin et al., 2003). Overlapping sense and antisense RNAs result in a high sequence complementarity. Thus, these two molecules could potentially anneal to each other, creating a dsRNA that can function as a perfect substrate for ADAR [and in the absence of ADAR, for MDA5, which can sense such structures and ignite an interferon response (Li et al., 2022)]. Despite several studies proposing such a mechanism (Kumar and Carmichael, 1998; Wang et al., 2000; Carmichael, 2003), only a few cases of editing in sense–antisense pairs have been reported to date (Kimelman and Kirschner, 1989; Peters et al., 2003; Athanasiadis et al., 2004; Li et al., 2022).

On the other hand, sense and antisense transcripts folding co-transcriptionally as independent domains can also generate distinct dsRNA without needing to pair with each other (Heilman-Miller and Woodson, 2003; Lai et al., 2013). dsRNA structures formed by local intramolecular interactions are in line with other reports on ADAR editing, showing that the majority of A-to-I antisense editing events are observed within *Alu* regions and only rarely within regions that could result from inter-molecular sense-antisense RNAs interactions (Athanasiadis et al., 2004; Neeman et al., 2005; Kawahara and Nishikura, 2006). Our observations with regard to *hLincRNA-p21*, where no modifications were observed outside the *Alu* regions, are in line with the hypothesis of dsRNA formed by the intramolecular interaction (Kawahara and Nishikura, 2006; Chillón and Pyle, 2016).

For the transcripts whose analysis motivated the work we report herein (*DDX58* and *hLincRNA-p21*), the antisense editing was catalyzed by ADAR1 (Figure 4). ADAR1-mediated editing represents most A-to-I editing in humans and occurs in non-coding regions of the transcriptome (Eisenberg and Levanon, 2018). The primary function of this editing is to discriminate between harmless endogenous (or “self”) and harmful exogenous viral dsRNAs, preventing activation of the cytosolic innate immune system in the absence of infection. Indeed, ADAR1-mediated editing of self dsRNA is required to avoid recognition of these structures by the dsRNA sensor melanoma differentiation-associated protein 5 (MDA5), which otherwise would bind self dsRNA and, upon interaction with the mitochondrial antiviral signaling protein (MAVS), would lead to an interferon response (Mannion et al., 2014; Liddicoat et al., 2015; Pestal et al., 2015). It is still not completely understood if specific self dsRNAs must be deaminated by ADAR1 to avoid the cytosolic innate immune reaction through MDA5. Recent work performed by JB Li and colleagues has shown that DNA mutations (SNPs) that culminate in a reduction of A-to-I editing within specific immunogenic dsRNAs underlie the risk for autoimmune and immune-related diseases. Notably, the authors identified two kinds of immunogenic dsRNAs, the ones that originated from an intramolecular pairing of inverted *Alu* repeats and, surprisingly, from an intermolecular pairing of antisense transcripts (Li et al., 2022).

Spurred by this finding, we performed a transcriptome-wide analysis looking for (annotated) antisense transcripts and matching them with reported editing events. Like others before us, we find that such events are rare overall. However, when convergent transcription overlaps with editing, at least a quarter (56 out of 199, ∼28%) of such transcripts are edited in the antisense orientation (thus generating apparent “U-to-C” RNA modification events). Around half of these are edited in both orientations, suggesting that these events, though rare, are not insignificant. It is important to note that antisense transcripts are frequently degraded by the nuclear RNA exosome limiting their detection in RNA-seq data. Using alternative NGS methods such as chromatin RNA-seq may improve the detection of antisense transcripts and, therefore, antisense editing. Finally, our analysis was limited to known antisense transcripts. Defining the antisense signal directly from the RNA-seq, despite being more challenging, may lead to the discovery of non-annotated antisense transcript and, thus, more antisense RNA editing.

Whether the antisense editing is derived from intra- or intermolecular interactions of RNAs, the fact that ADAR1 edits overlapping sense and antisense RNAs may suggest those transcripts as particularly relevant in activating MDA5 and, therefore, could be highly immunogenic. In such a scenario, the identification of clusters of apparent U-to-C and A-to-I modifications could simplify the prediction of potentially strongly immunogenic self-dsRNAs [which are thought to be functionally relevant (Li et al., 2022)].

It is also interesting to notice that changes in sense and antisense RNA editing upon treatments may happen for different reasons. For example, the decrease in antisense editing within *DDX58* upon IFN treatment is probably due to the ∼20-fold increase in expression of its sense-transcript together with a 2-fold increase in ADAR1 expression (Supplementary Figure S1). Intriguingly, upon doxorubicin treatment, we observed an increase in both sense and antisense editing without any increase in ADAR1 expression (Figure 3 and Supplementary Figure S3B). These results are in agreement with recent findings by Huertas and others, which describe an increase of A-to-I editing upon treatment with DSBs-inducing agents, despite no changes in ADAR protein expression levels (Jimeno et al., 2021).

Regarding *DDX58*, it is interesting to note that although the locus is not annotated as a source of cis-NATs, we can functionally identify antisense transcripts in some cell lines (RCK8, U2932, HEK293T) but not in others (e.g., A549). Indeed, A549 shows abundant A-to-I but no U-to-C editing indicating the absence of antisense transcription (Figure 1). Considering that *DDX58* is an ISG and its transcription is highly regulated (Matsumiya and Stafforini, 2010), it is tempting to speculate that antisense transcription from the *DDX58* locus could have regulatory functions.

Overall, our study demonstrates that antisense A-to-I editing can result in instances of apparent U-to-C RNA modification, which may be misinterpreted as novel modification events. At the same time, we note that clusters of A-to-I and “U-to-C” modification events could be simple markers of ADAR activity on functionally important loci (a characteristic that will aid in their identification).

## Data Availability

Publicly available datasets were analyzed in this study. This data can be found here: The RNA datasets analyzed in this study can be downloaded from Gene Expression Omnibus at NCBI under the accessions GSM4118041, GSM4118068, GSM4118074, GSM4118077, GSM4118080, GSM4118083, GSM4118086, respectively for samples RNAAtlas285, RNAAtlas294, RNAAtlas296, RNAAtlas297, RNAAtlas298, RNAAtlas299 and RNAAtlas300. Computer code used for the A-to-I sense-antisense RNA editing analysis can be found at https://github.com/BioinfoUNIBA/antisenseEditing.

## Supplementary material

Supplementary Material and information about editing sites in the NGS data can be found in Data Presentation 1 and Table 1, respectively.
