# Supplementary material for: ADAR RNA editing on antisense RNAs results in apparent U-to-C base changes on overlapping sense transcripts
Source: Front Cell Dev Biol. 2023 Jan 6;10:1080626. doi: 10.3389/fcell.2022.1080626 (PMC9852825; doi:10.3389/fcell.2022.1080626)
Supplement: Supplementary file 2 [file Presentation1.pdf]

Supplementary Material

1 Supplementary Figures and Tables

1.1 Supplementary Tables

#	*Name*	*Sequence*	*Purpose*	*Figure*
1	DDX58 ed F	ATTTGGCCCTGTTGAGCACTCT	To check editing on DDX58	1, 2, 4, S2
2	DDX58 ed R	ACGTCCAGGAAACCGCAAACTA	To check editing on DDX58	1, 4
3	DDX58 pJet R	GAGAACAATGGCACACGTTAAGAG	To check editing on DDX58 in pJet	2, S2
4	hLincRNA-p21 F	GACACAGGAGAGGCAAGATAG	To check editing on hLincRNA-p21 sense Alu	3, 4, S4
5	hLincRNA-p21 R	CGGGCCTCTGTAAGTATGAAC	To check editing on hLincRNA-p21 sense Alu	3, 4, S4
6	DDX58 qPCR F	GGTATAGAGTTACAGGCATTTC	To check expression of DDX58	S1
7	DDX58 qPCR R	TTGTTTACTAGTGTTGTGGC	To check expression of DDX58	S1
8	hLincRNA-p21 qPCR F	GTCTACCTGCTGCTCTAGGA	To check expression of hLincRNA-p21	S3
9	hLincRNA-p21 qPCR R	CTGTTCTCAGAGCCACTTGTC	To check expression of hLincRNA-p21	S3
10	PGK1 qPCR F	CTAAGCAGATTGTGTGGAATG	Housekeeping gene for qPCR	S1, S3
11	PGK1 qPCR R	CTCACATGGCTGACTTTATC	Housekeeping gene for qPCR	S1, S3
12	sgRNA F exon 4	caccGGACAGGAGACGGAATTCGC	To clone sgRNA ADAR1 exon 4 in LentiCRISPRv2	S7
13	sgRNA R exon 4	aaacGCGAATTCCGTCTCCTGTCC	To clone sgRNA ADAR1 exon 4 in LentiCRISPRv2	S7
14	non-targeting sgRNA F	caccGTATTACTGATATTGGT	To clone a non-targeting sgRNA in LentiCRISPRv2	S7
15	non-targeting sgRNA R	aaacACCAATATCAGTAATAC	To clone a non-targeting sgRNA in LentiCRISPRv2	S7
16	sgRNA DTWD1	tatatatcttgtggaaaggacgaaacaccGCTTCGTTTGAGAAATATAGGgtttaagagctatgctggaaacagcatag	To clone exon 2 sgRNA DTWD1 in pSpCas9(BB)-2A-GFP	S5
17	sgRNA DTWD2	tatatatcttgtggaaaggacgaaacaccGATGCTGAATTATGTACAAGgtttaagagctatgctggaaacagcatag	To clone exon 2 sgRNA DTWD2 in pSpCas9(BB)-2A-GFP	S5
18	sgRNA TSR3	tatatatcttgtggaaaggacgaaacaccGAGGCTGGACGAGACACCGTTgtttaagagctatgctggaaacagcatag	To clone exon 3 sgRNA TSR3 in pSpCas9(BB)-2A-GFP	S5
19	non-targeting sgRNA	tatatatcttgtggaaaggacgaaacaccGTATTACTGATATTGGTgtttaagagctatgctggaaacagcatag	To clone a non-targeting sgRNA in pSpCas9(BB)-2A-GFP	S5
20	DTWD1 ko F	AGTGTGCCAATTTGGTGGTT	To screen DTWD1 ko clones	S5
21	DTWD1 ko R	TCCCACTTTGCTGAGCTTTT	To screen DTWD1 ko clones	S5
22	DTWD2 ko F	CCTCTTTGTATAAATGCAGTGGGATTAAGGTC	To screen DTWD2 ko clones	S5
23	DTWD2 ko R	CTTCGTGTTTCCTCTTGTGGACTC	To screen DTWD2 ko clones	S5
24	TSR3 ko F	AGGTCGTCTTCATTTCCAGC	To screen TSR3 ko clones	S5

25	TSR3 ko R	GCAGGAAAGTCTGTAGGGC	To screen TSR3 ko clones	S5
26	rRNA18S 1248 F	GTTCGAAGACGATCAGATACCG	To check U-to-C in rRNA18S, position 1248	S5
27	rRNA18S 1248 R	TCGTTCGTTATCGGAATTAACCAG	To check U-to-C in rRNA18S, position 1248	S5
28	ADAR1 qPCR F	CCCTTCAGCCACATCCTTC	To check expression of ADAR1	S1, S3
29	ADAR1 qPCR R	GCCATCTGCTTTGCCACTT	To check expression of ADAR1	S1, S3

Supplementary Table 1 | DNA oligos used in this study. Oligos from #1-11 and #28-29 were used for PCR and RT-PCR amplification, sequencing, and qRT-PCR analysis. Oligos #12-15 were used to generate Lenti-CRISPR-ADAR1 or lenti-CRISPR-NT. Oligos #20-29 were used for the generation and the screening of DTWD1, DTWD2 and TSR3 knockout cell lines. Lowercase letters represent the homology part needed for cloning.

Chromosomal location (hg19)	***Ref***	***Ed***	***Transcript***	***Strand***	***Figure***
Chr9:32456486	T	C	*DDX58*	-	1, 2, 4
Chr9:32456467	A	G	*DDX58*	-	1, 2, 4
Chr9:32456453	T	C	*DDX58*	-	1, 2, 4
Chr9:32456449	T	C	*DDX58*	-	1, 2, 4
Chr9:32456433	A	G	*DDX58*	-	1, 2, 4
Chr9:32456413	T	C	*DDX58*	-	1, 2, 4
Chr9:32456380	T	C	*DDX58*	-	1, 2, 4
Chr9:32456376	A	G	*DDX58*	-	1, 2, 4
Chr9:32456371	T	C	*DDX58*	-	1, 2, 4
Chr9:32456369	A	G	*DDX58*	-	1, 2, 4
Chr9:32456368	T	C	*DDX58*	-	1, 2, 4
Chr9:32456367	T	C	*DDX58*	-	1, 2
Chr9:32456365	T	C	*DDX58*	-	1, 2, 4
Chr9:32456355	A	G	*DDX58*	-	1, 2, 4
Chr9:32456353	A	G	*DDX58*	-	1, 2, 4
Chr9:32456318	T	C	*DDX58*	-	1, 2, 4
Chr9:32456316	T	C	*DDX58*	-	1, 2, 4
Chr9:32456306	A	G	*DDX58*	-	1, 2, 4
Chr9:32456305	A	G	*DDX58*	-	1, 2, 4
Chr9:32456304	A	G	*DDX58*	-	1, 2, 4
Chr9:32456303	A	G	*DDX58*	-	1, 2, 4
Chr9:32456291	A	G	*DDX58*	-	1, 2, 4
Chr6:36,632,601	T	C	*hLincRNA-p21*	-	3
Chr6:36,632,600	T	C	*hLincRNA-p21*	-	3
Chr6:36,632,588	A	G	*hLincRNA-p21*	-	3
Chr6:36,632,575	T	C	*hLincRNA-p21*	-	3
Chr6:36,632,573	T	C	*hLincRNA-p21*	-	3
Chr6:36,632,572	T	C	*hLincRNA-p21*	-	3
Chr6:36,632,565	T	C	*hLincRNA-p21*	-	3
Chr6:36,632,549	T	C	*hLincRNA-p21*	-	3
Chr6:36,632,538	T	C	*hLincRNA-p21*	-	3
Chr6:36,632,536	T	C	*hLincRNA-p21*	-	3
Chr6:36,632,491	T	C	*hLincRNA-p21*	-	3
Chr6:36,632,486	T	C	*hLincRNA-p21*	-	3
Chr6:36,632,485	T	C	*hLincRNA-p21*	-	3
Chr6:36,632,456	T	C	*hLincRNA-p21*	-	3, 4
Chr6:36,632,447	T	C	*hLincRNA-p21*	-	3
Chr6:36,632,445	A	G	*hLincRNA-p21*	-	3, 4
Chr6:36,632,439	T	C	*hLincRNA-p21*	-	3
Chr6:36,632,429	T	C	*hLincRNA-p21*	-	3, 4

Chr6:36,632,420	T	C	*hLincRNA-p21*	-	3
Chr6:36,632,418	A	G	*hLincRNA-p21*	-	3, 4
Chr6:36,632,417	A	G	*hLincRNA-p21*	-	3, 4
Chr6:36,632,415	T	C	*hLincRNA-p21*	-	3
Chr6:36,632,410	T	C	*hLincRNA-p21*	-	3, 4
Chr6:36,632,401	T	C	*hLincRNA-p21*	-	3, 4
Chr6:36,632,399	T	C	*hLincRNA-p21*	-	3
Chr6:36,632,396	T	C	*hLincRNA-p21*	-	3
Chr6:36,632,387	T	C	*hLincRNA-p21*	-	3
Chr6:36,632,386	T	C	*hLincRNA-p21*	-	3
Chr6:36,632,384	T	C	*hLincRNA-p21*	-	3, 4
Chr6:36,632,383	T	C	*hLincRNA-p21*	-	3, 4
Chr6:36,632,377	T	C	*hLincRNA-p21*	-	3, 4
Chr6:36,632,376	T	C	*hLincRNA-p21*	-	3, 4
Chr6:36,632,372	A	G	*hLincRNA-p21*	-	3, 4
Chr6:36,632,366	A	G	*hLincRNA-p21*	-	3, 4
Chr6:36,632,363	A	G	*hLincRNA-p21*	-	3, 4
Chr6:36,632,362	T	C	*hLincRNA-p21*	-	3
Chr6:36,632,358	T	C	*hLincRNA-p21*	-	3
Chr6:36,632,356	T	C	*hLincRNA-p21*	-	3
Chr6:36,632,352	T	C	*hLincRNA-p21*	-	3
Chr6:36,632,351	T	C	*hLincRNA-p21*	-	3
Chr6:36,632,350	T	C	*hLincRNA-p21*	-	3
Chr6:36,632,348	A	G	*hLincRNA-p21*	-	3, 4
Chr6:36,632,347	T	C	*hLincRNA-p21*	-	3
Chr6:36,632,338	A	G	*hLincRNA-p21*	-	3
Chr6:36,632,337	T	C	*hLincRNA-p21*	-	3, 4
Chr6:36,632,335	A	G	*hLincRNA-p21*	-	3
Chr6:36,632,327	T	C	*hLincRNA-p21*	-	3
Chr6:36,632,322	A	G	*hLincRNA-p21*	-	3, 4
Chr6:36,632,321	T	C	*hLincRNA-p21*	-	3, 4
Chr6:36,632,320	T	C	*hLincRNA-p21*	-	3, 4
Chr6:36,632,315	T	C	*hLincRNA-p21*	-	3
Chr6:36,632,312	A	G	*hLincRNA-p21*	-	3, 4
Chr6:36,632,311	T	C	*hLincRNA-p21*	-	3, 4
Chr6:36,632,304	T	C	*hLincRNA-p21*	-	3
Chr6:36,632,300	A	G	*hLincRNA-p21*	-	3, 4
Chr6:36,632,298	T	C	*hLincRNA-p21*	-	3
Chr6:36,632,294	T	C	*hLincRNA-p21*	-	3
Chr6:36,632,289	T	C	*hLincRNA-p21*	-	3
Chr6:36,632,288	T	C	*hLincRNA-p21*	-	3

Chr6:36,632,283	A	G	*hLincRNA-p21*	-	3, 4
Chr6:36,632,279	T	C	*hLincRNA-p21*	-	3
Chr6:36,632,272	T	C	*hLincRNA-p21*	-	3
Chr6:36,632,268	T	C	*hLincRNA-p21*	-	3, 4
Chr6:36,632,262	A	G	*hLincRNA-p21*	-	3
Chr6:36,632,259	T	C	*hLincRNA-p21*	-	3
Chr6:36,632,255	T	C	*hLincRNA-p21*	-	3
Chr6:36,632,252	A	G	*hLincRNA-p21*	-	3, 4
Chr6:36,632,247	A	G	*hLincRNA-p21*	-	3, 4
Chr6:36,632,244	A	G	*hLincRNA-p21*	-	3, 4
Chr6:36,632,238	A	G	*hLincRNA-p21*	-	3, 4
Chr6:36,632,237	T	C	*hLincRNA-p21*	-	3
Chr6:36,632,233	A	G	*hLincRNA-p21*	-	3
Chr6:36,632,218	T	C	*hLincRNA-p21*	-	3, 4

Supplementary Table 2 | Chromosomal locations of all the editing sites analyzed in this study. In green and red are highlighted locations resulting in A-to-I and U-to-C base changes, respectively. Ref = reference; Ed = edited.

1.2 Supplementary Figures

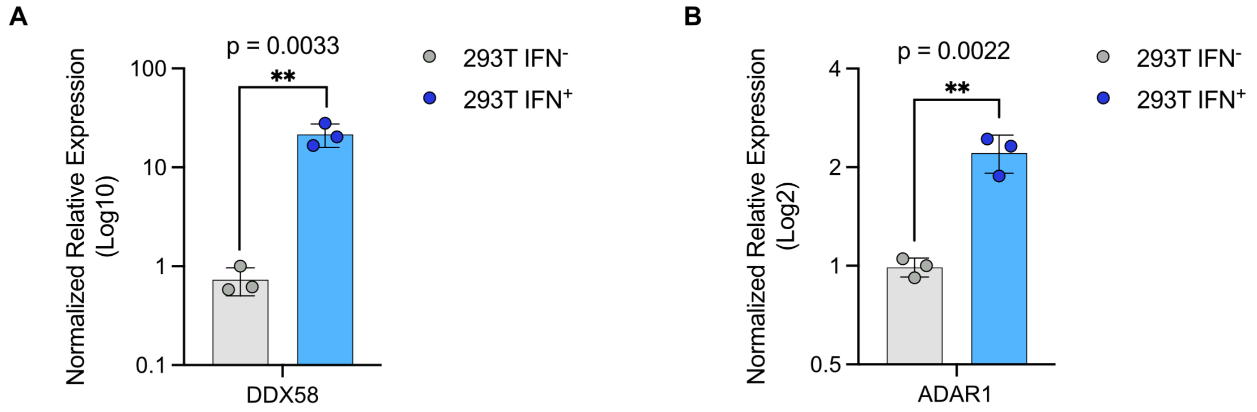

Supplementary Figure 1 | Interferon α treatment leads to an increase in *DDX58* (**A**) and *ADAR1* (**B**) expression. The bar plot represents normalized relative mRNA expression measured by qPCR in HEK293T cells upon IFNα stimulation. The expression was normalized on IFNα negative samples. *PGK1* was always used as a housekeeping gene. A two-tailed unpaired t-test was used to compare the differences. **$p<0.01$. Center = mean and error bars = standard deviation, N = 3.

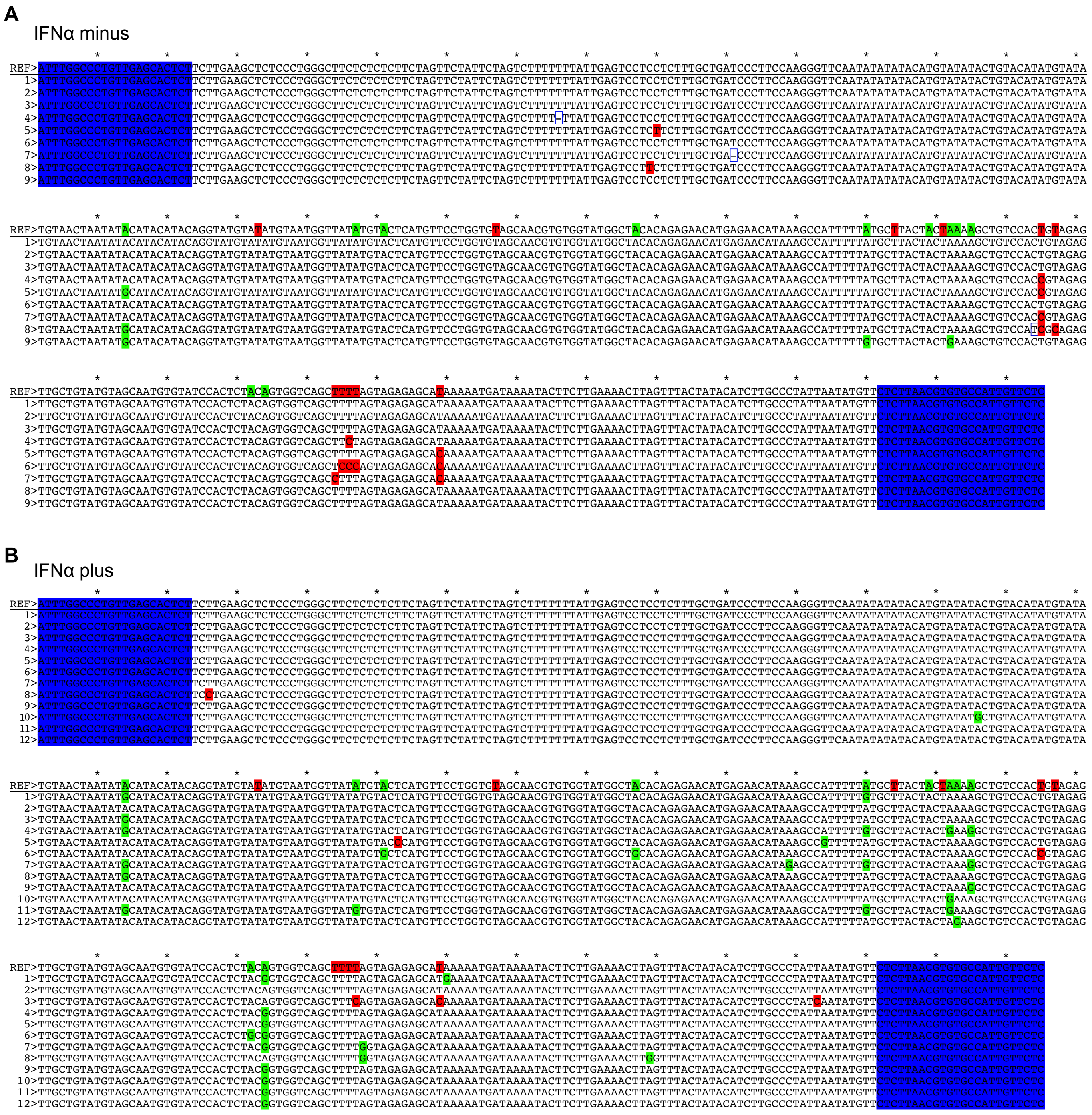

Supplementary Figure 2 | U-to-C base changes within *DDX58* upon interferon α treatment. **(A-B)** Alignment of sequences obtained from single bacterial colonies. The first line is the genomic reference. The sequence highlighted in blue represents the sequence bound by the primers used during amplification. A-to-Gs (corresponding to A-to-I) are highlighted in green, T-to-Cs (corresponding to U-to-C) in red, and any other change with empty blue rectangles. **A** shows the alignment of sequences without IFNα, while **B** shows the alignment upon IFNα stimulation.

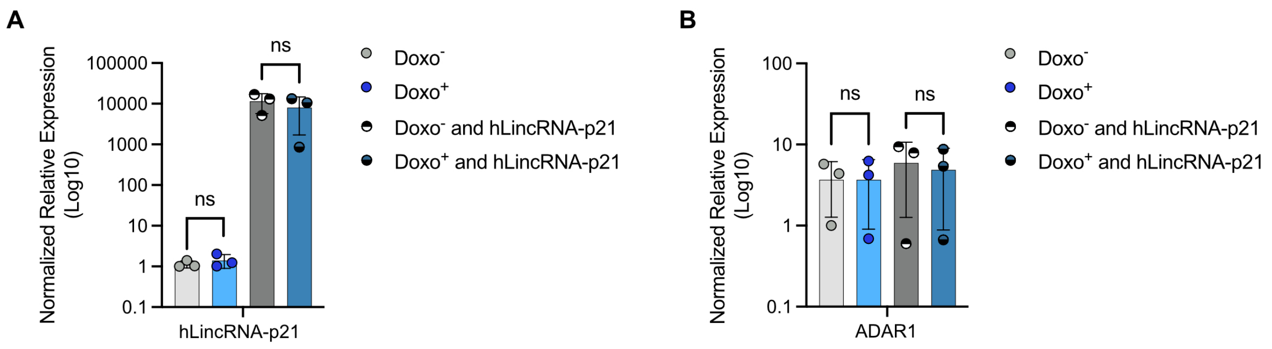

Supplementary Figure 3 | Doxorubicin treatment doesn't affect *hLincRNA-p21* expression, stability, or *ADAR1* expression. The bar plot represents the normalized relative expression of *hLincRNA-p21* (**A**) and *ADAR1* (**B**) measured by qPCR. The expression was normalized on doxorubicin-negative samples without *hLincRNA-p21* overexpression (samples in light grey). *PGK1* was always used as a housekeeping gene. A two-tailed unpaired t-test was used to compare the differences. ns = not significant. Center = mean and error bars = standard deviation, N = 3.

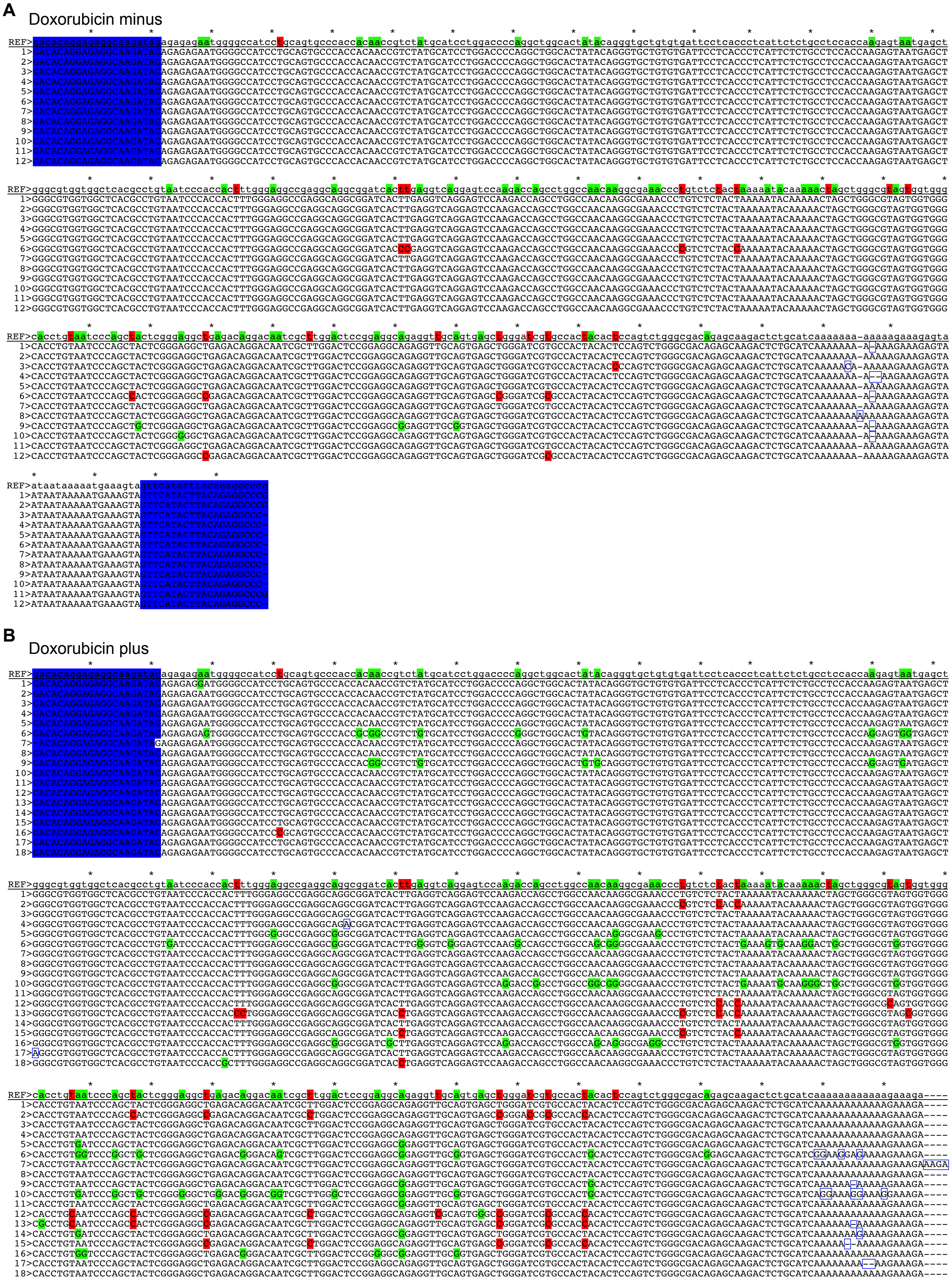
A
Doxorubicin minus
B
Doxorubicin plus

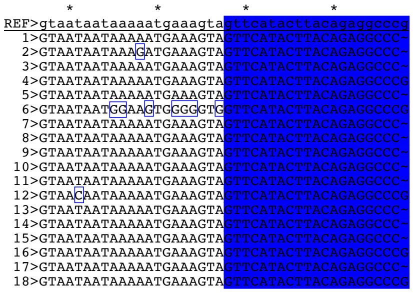

Supplementary Figure 4 | U-to-C base changes within *hLincRNA-p21* upon doxorubicin treatment. **(A-B)** Alignment of sequences obtained from single bacterial colonies. The first line is the plasmid reference. The sequence highlighted in blue represents the sequence bound by the primers used during amplification. A-to-Gs (corresponding to A-to-I) are highlighted in green, T-to-Cs (corresponding to U-to-C) in red, and any other change with empty blue rectangles. **A** shows the alignment of sequences without doxorubicin, while **B** shows the alignment upon doxorubicin treatment.

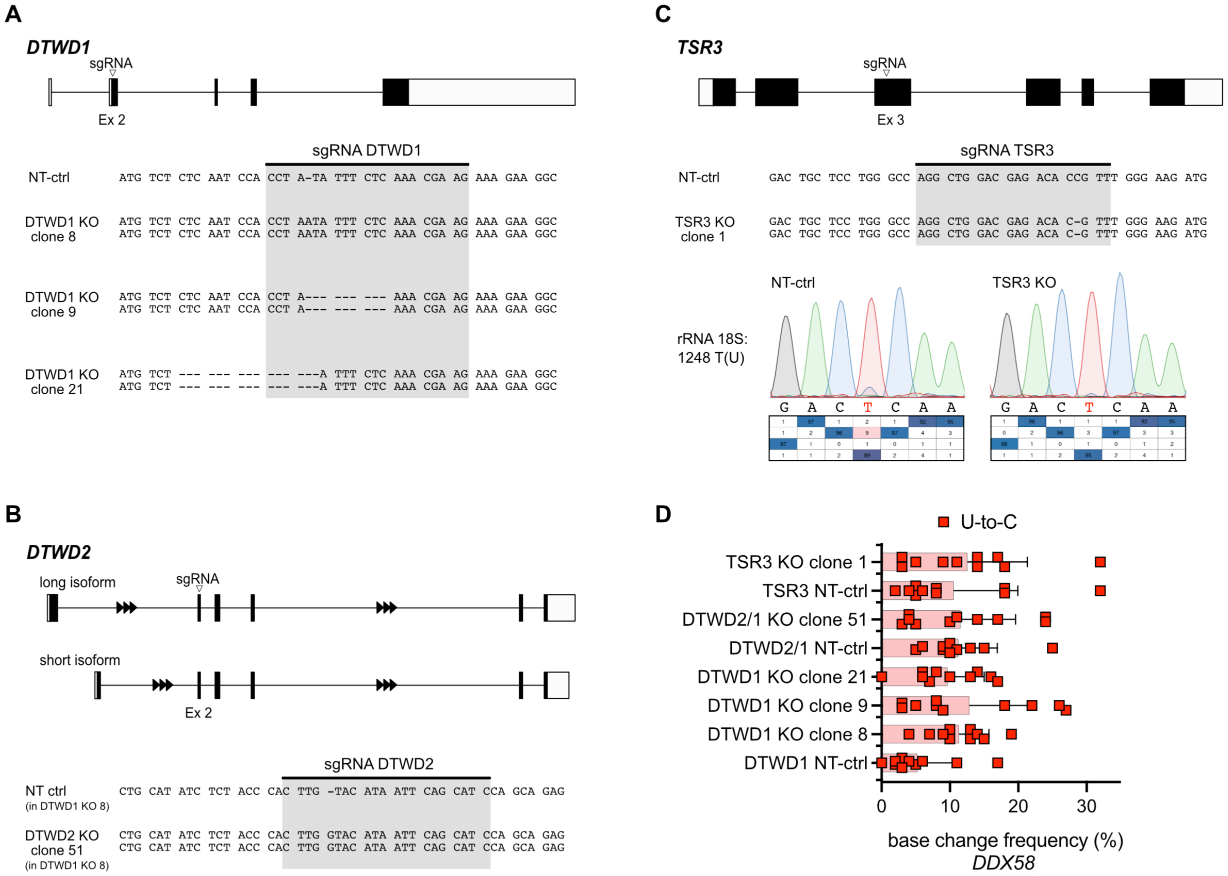

Supplementary Figure 5 | DTWD1, DTWD2, and TSR3 knockout cell lines. **(A, B)** Diagram of the human DTWD1 and DTWD2 genes and sgRNAs used (upper). The lower part of these panels shows the genotypes of the knockout clones. DTWD2 KO was performed in DTWD1 KO clone 8 to obtain a double knockout for DTWD1 and DTWD2. **(C)** TSR3 gene and the sgRNA used (upper). Genotypes of the TSR3 knockout clone (middle). The lower part of this panel shows the functional validation of the TSR3 KO. Indeed, the decrease of U-to-C base-change at nucleotide 1248. U of the rRNA 18S is due to the loss of m1acp3ψ. **(D)** U-to-C frequency within *DDX58* 3'UTR in the different KO cell lines.

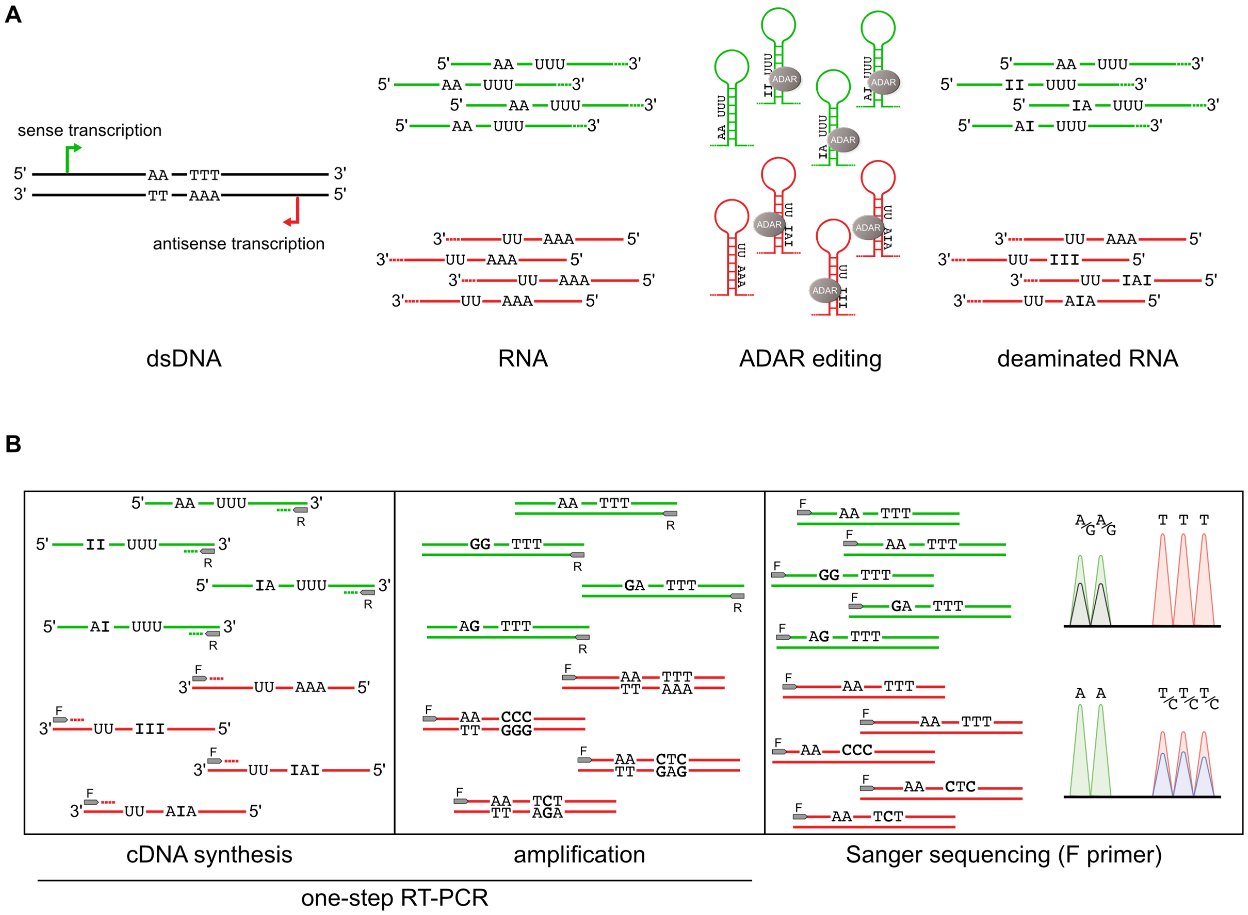

Supplementary Figure 6 | The rationale behind the apparent U-to-C editing deriving from A-to-I RNA editing on antisense RNA. **(A)** Schematic representing A-to-I RNA editing mediated by ADAR on the sense (in green) and antisense (in red) RNAs. **(B)** Schematic to explain the molecular details of how during one-step RT-PCR, cDNA is synthesized from both sense (in green) and antisense (in red) RNAs, leading to T-to-C base changes after DNA amplification. Sanger sequencing using the forward primer results in A-to-G or T-to-C double picks for A-to-I RNA editing of sense and antisense RNA, respectively.

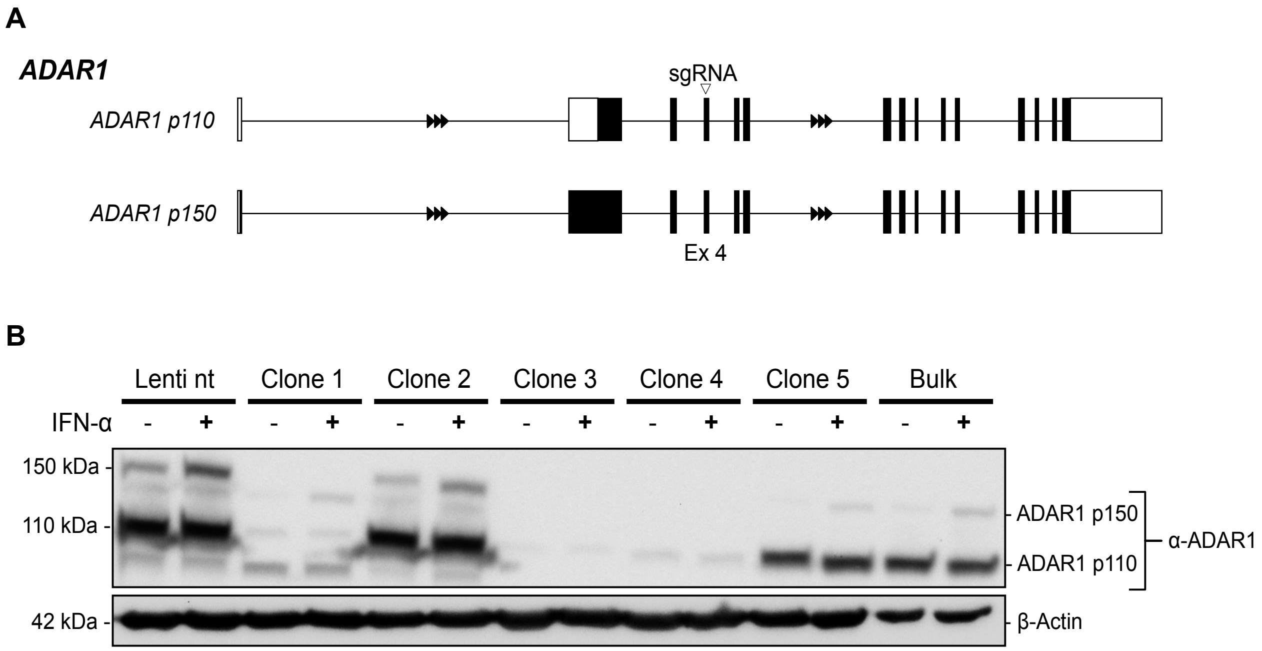

Supplementary Figure 7 | HEK293T ADAR1 knockout cell line. **(A)** Diagram of the human ADAR1 gene, with the two isoforms p110 and p150 and the exon targeted by the sgRNA. **(B)** Western blot of HEK293T KO clones and bulk with or without IFN-α stimulation. Clone 3 is the one used in this study.

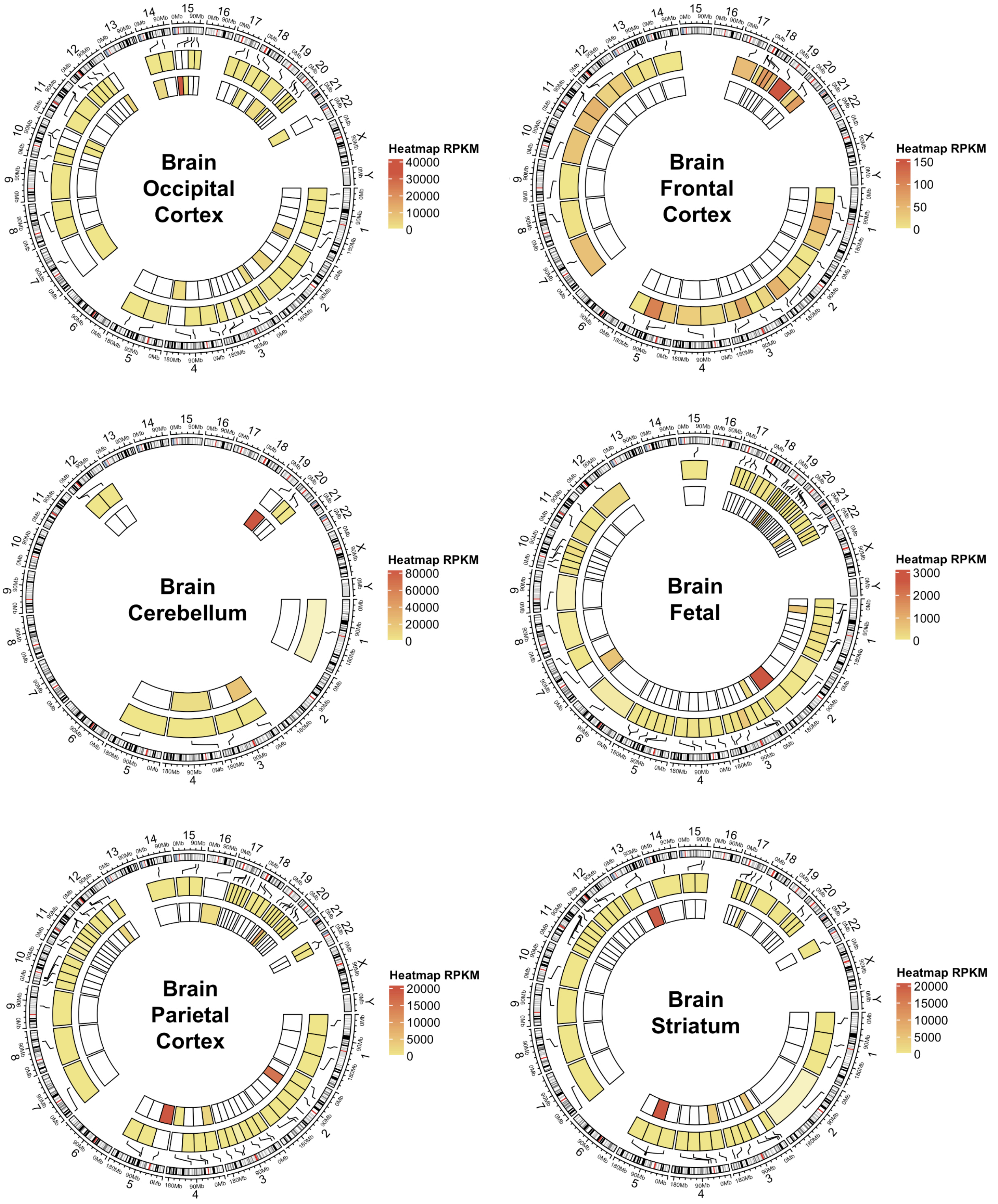

Supplementary Figure 8 | Antisense RNA editing in different human tissues. Circular heatmaps for RNAseq samples from several human tissues. For each tissue, the external circle represents the chromosomes, and the two inner circles represent sense (intermediate circle) and antisense (internal circle) editing. A-to-I RNA editing locations are indicated with black lines connecting the heatmap to the cytoband context of the chromosomes (human genome assembly hg38). Editing levels are depicted in circular heatmaps using a color scale based on RPKM-like values.
